# Statins Affect the Presentation of Endothelial Chemokines by Targeting to Multivesicular Bodies

**DOI:** 10.1371/journal.pone.0040673

**Published:** 2012-07-16

**Authors:** Johanna Hol, Kari Otterdal, Unni M. Breland, Espen Stang, Turid M. Pedersen, Kathrine Hagelsteen, Trine Ranheim, Monika Kasprzycka, Bente Halvorsen, Guttorm Haraldsen, Pål Aukrust

**Affiliations:** 1 Division of Pathology, Oslo University Hospital, Oslo, Norway; 2 Research Institute for Internal Medicine, Oslo University Hospital, Oslo, Norway; University of Leuven, Rega Institute, Belgium

## Abstract

**Background:**

In addition to lowering cholesterol, statins are thought to beneficially modulate inflammation. Several chemokines including CXCL1/growth-related oncogene (GRO)-α, CXCL8/interleukin (IL)-8 and CCL2/monocyte chemoattractant protein (MCP)-1 are important in the pathogenesis of atherosclerosis and can be influenced by statin-treatment. Recently, we observed that atorvastatin­treatment alters the intracellular content and subcellular distribution of GRO-α in cultured human umbilical vein endothelial cells (HUVECs). The objective of this study was to investigate the mechanisms involved in this phenomenon.

**Methodology/ Principal Findings:**

The effect of atorvastatin on secretion levels and subcellular distribution of GRO-α, IL-8 and MCP-1 in HUVECs activated by interleukin (IL)-1β were evaluated by ELISA, confocal microscopy and immunoelectron microscopy. Atorvastatin increased the intracellular contents of GRO-α, IL-8, and MCP-1 and induced colocalization with E-selectin in multivesicular bodies. This effect was prevented by adding the isoprenylation substrate GGPP, but not the cholesterol precursor squalene, indicating that atorvastatin exerts these effects by inhibiting isoprenylation rather than depleting the cells of cholesterol.

**Conclusions/ Significance:**

Atorvastatin targets inflammatory chemokines to the endocytic pathway and multivesicular bodies and may contribute to explain the anti-inflammatory effect of statins at the level of endothelial cell function.

## Introduction

Cardiovascular disease is the leading cause of death worldwide, and atherosclerosis is one of its major underlying causes [1]. A clear correlation has been established between elevated plasma cholesterol and atherosclerotic disease, but compelling evidence suggests that inflammation also plays an important role in atherogenesis [1], [2].

Statins are cholesterol-lowering agents that inhibit 3-hydroxy-3-methyl-glutaryl-CoA (HMG CoA) reductase, the rate-limiting enzyme in the mevalonate synthesis pathway. Mevalonate is essential for the synthesis of cholesterol, sterol and bile acids, but is also of major importance for isoprenylation of proteins [3], [4], [5], [6]. In addition to reducing serum cholesterol levels, statins have anti-inflammatory, immunomodulatory and anti-thrombotic effects and are able to improve endothelial dysfunction, potentially contributing to their favorable effects in atherosclerosis [5]. Lipid reduction can in itself be anti-inflammatory and improve endothelial function, but the pleiotropic effects of statins appear to also involve cholesterol-independent mechanisms [3].

Migration of leukocytes such as monocytes and T cells to atherosclerotic lesions is a crucial step in atherogenesis, initiated by the endothelial surface expression of adhesion molecules including selectins, ligands of G protein-coupled receptors (GPCRs) and integrin ligands [7], [8]. Although several ligands with relevance to atherogenesis may activate GPCRs, chemokines have a particular role in regulating leukocyte traffic into tissues, as the different subsets of leukocytes express characteristic chemokine receptor profiles [8]. Indeed, the chemokines growth-related oncogene (GRO)-α/CXCL1 interleukin (IL)-8/CXCL8 and monocyte chemoattractant protein (MCP)-1/CCL2 have all been implicated in the pathogenesis of atherosclerosis [9], [10], [11], at least partly through their ability to attract and activate leukocytes into the vessel wall.

Statins may reduce leukocyte rolling and adhesion to inflamed endothelium [12] and the infiltration of leukocytes to inflammatory lesions [13], [14] by affecting both the endothelial surface translocation of P-selectin [12], [13] and the interactions between leukocyte integrins and their endothelial ligands [15]. Statins may also interfere with chemokine transcription by inhibition of histone acetylation and phosphorylation, reducing binding of transcription factors like NF-κB to chemokine promoters [16], [17]. As mevalonate and its metabolites are involved in a variety of intracellular processes, statins also have the potential to affect chemokine expression on many other stages, including synthesis, intracellular trafficking and rate of degradation [6]. We recently described that ortho-hydroxy-atorvastatin, a metabolite of atorvastatin, mediates intracellular accumulation and alters subcellular distribution of GRO-α in human umbilical vein endothelial cells (HUVECs). In the present study we demonstrate that the related chemokines IL-8 and MCP-1 are affected in the same manner, and that the intracellular accumulation is due to the occurrence of chemokines in multivesicular bodies (MVBs), suggesting that they may be destined for lysosomal degradation.

## Methods

### Reagents

Ortho-hydroxy-atorvastatin (here referred to as atorvastatin) was a gift from Pfizer (New York, NY). Recombinant human epidermal growth factor (EGF), basic fibroblast growth factor (bFGF), IL-1β, interferon (IFN)-γ and tumor necrosis factor (TNF)-α were from R&D Systems (Abingdon, UK). MCDB 131 and Opti-MEM I medium, fetal calf serum (FCS), gentamicin, fungizone and L-glutamine were from Invitrogen Life Sciences (Paisley, UK) and trypsin-EDTA (ethylenediaminetetraacetic acid) from Bio-Whittaker (Walkerswille, MD). Restriction enzymes were from New England Biolabs (Hitchin, UK). Unless otherwise noted, all other reagents including simvastatin, fluvastatin and pravastatin were from Sigma-Aldrich (St. Louis, MO).

### Cell Culture

Umbilical cords were obtained from the Department of gynaecology and obstetrics, Rikshospitalet. They were used with the motherś written permission and the study protocol was specifically approved by the Regional Committee for Medical Research Ethics (Health Region South, Norway, Approval S- 05152). HUVECs were isolated as described by Jaffe et al [18] and cultured in MCDB 131 containing 7.5% FCS, 10 ng/ml EGF, 1 ng/ml bFGF, 2 mM L-glutamine, 1 µg/ml hydrocortisone, 50 µg/ml gentamicin, and 250 ng/ml fungizone. The cells were maintained at 37°C in a humid 95% air/ 5% CO_2_ atmosphere, split at a ratio of 1∶3 and used at passage levels 2–6. Human aortic endothelial cells (HAoECs) were purchased (C-12271, Promocell, Heidelberg, Germany), cultured in custommade medium (Promocell Endothelial Cell Growth Medium MV2) as above and used in passage 5.

### Secretion Experiments

HUVECs were seeded (1.6×10^4^ cells/well) in 96-well plates (BD Biosciences, San Jose, CA) and cultured to confluence before pre-treatment for 2 hours with atorvastatin and/ or substrates of the mevalonate pathway (mevalonate 1 mM, squalene 1 mM or geranylgeranyl pyrophosphate [GGPP] 100 µM) followed by stimulation with IL-1β (5 ng/ml) for 20–22 hours. Supernatants were harvested, and cells were washed in cold PBS before lysis in lysis buffer (Nonidet P-40 1% in 150 mM NaCl, 50 mM Tris HCl, pH 7.8) and addition of proteinase inhibitors (Sigma P8340, 1∶100). Materials were stored at −70°C until the time of analysis.

### Enzyme-linked Immunoassay (ELISA)

The chemokines (GRO-α MCP-1 and IL-8) were analyzed by DuoSet ELISA kits or matched antibody pairs (R&D Systems or Peprotech, Rocky Hill, NJ) like previously described [19].

### Cell Detachment and Apoptosis

Cell detachment was quantified in cells from secretion experiments by fixation in 4% PFA and staining with 0.1% crystal violet. The cells were washed under running water and the dye was dissolved in 33% acetic acid. Absorbance at 550 nm was measured by a Tecan Sunrise Microplate reader (Tecan Austria Gesellschaft, Grödig, Austria). Relative quantification of histone-complexed DNA was performed with the Cell Death Detection ELISA^PLUS^ Kit as described by the manufacturers (Roche Diagnostics, Indianapolis, IN), using camptothecin as a positive control. The apoptosis assay was performed once, using cells from two individual donors.

### Immunostaining, Microscopy, Electron Microscopy and Evaluation of Images

HUVECs or HAoECs were seeded in gelatin-coated 8-well LabTek™ chamber slides (Nalge Nunc International, Rochester, NY), pre-treated with atorvastatin for 2 hours, stimulated with IL-1β, TNF-α or LPS and cultured for 20–22 hours before fixation with 4% paraformaldehyde. For immunofluorescence slides were incubated with primary antibodies for 16–20 hours at 4°C, washed in PBS containing 0.1% saponin, incubated with biotinylated secondary antibodies for 90 minutes at room temperature, washed, incubated with streptavidin-Cy3 for 60 minutes, washed, dipped in distilled H_2_O, dried and mounted in polyvinyl alcohol (PVA). For costaining, Alexa488-conjugated secondary antibodies were added to the last two incubations. All antibodies were diluted in PBS containing 1.25% BSA and 0.1% saponin. Images were obtained using a Leica TSC XP confocal microscope (Leica Microsystems, Heidelberg, Germany) equipped with an Ar (488 nm) and two He/Ne (543 and 633 nm) lasers and PL Apo 40×/1.25–0.75 and N Plan apochromat 100×/1.4 oil objectives. For electron microscopy cells were fixed with 4% paraformaldehyde and 0.1% glutaraldehyde in Sorensen’s phosphate buffer and processed for cryosectioning and immunolabeling as described [20]. Bound antibodies were visualized using protein A gold (Cell Microscopy Center, Utrecht, The Netherlands). The sections were examined using a Tecnai Spirit electron microscope (FEI Company, Hillsboro, OR) equipped with a Morada digital camera (Olympus Soft Imaging Solutions GmbH, Muenster, Germany). All images were processed using Adobe Photoshop (CS2, CS4, CS5). Details of antibodies used are given in table 1.

**Table 1:** Antibodies used for immunostainings.

Specificity	Specification	Working conc.	Source (Product no.)
GRO-α	Rabbit polyclonal	2 µg/ml	Peprotech (500-P92)
GRO-α	Mouse IgG_1_	5 µg/ml	R&D Systems (MAB275)
IL-8	Mouse IgG_1_	2 µg/ml	Peprotech (500-M08)
MCP-1	Mouse IgG_2B_	2 µg/ml	R&D Systems (MAB679)
E-selectin	Mouse IgG_1_	1 µg/ml	Becton Dickinson (550023)
EEA-1	Mouse IgG_1_	1.2 µg/ml	BD Transduction Laboratories (610457)
CD63	Mouse IgG_1_	2 µg/ml	DSHB, University of Iowa (clone H5C6)
VWF	Rabbit polyclonal	1/2000	DAKO (A0082)

### Statistics

Histograms show pools of results from three individual experiments with HUVECs from different donors. Error bars show standard errors of the mean. Prism 5 for Mac OS × (GraphPad Software Inc., La Jolla, CA) was used to calculate means, standard errors and significances using the student t-test or one-way ANOVA and Bonferroni testing. P values are two-sided and considered significant when <0.05.

## Results

We have recently shown that atorvastatin impairs secretion of the chemokine GRO-α from IL-1β-stimulated HUVECs [11]. To investigate if this is also the case for other chemokines implicated in atherosclerosis and to explore the mechanism, we used IL-1β-stimulated HUVECs pretreated with increasing doses of atorvastatin. Chemokine levels in supernatants and lysates were measured by ELISA, and the subcellular localization of chemokines was visualized by immunofluorescent staining and immunoelectron microscopy.

### Atorvastatin Increases Intracellular Levels of IL-1β-induced Endothelial Chemokines

**Figure 1:**
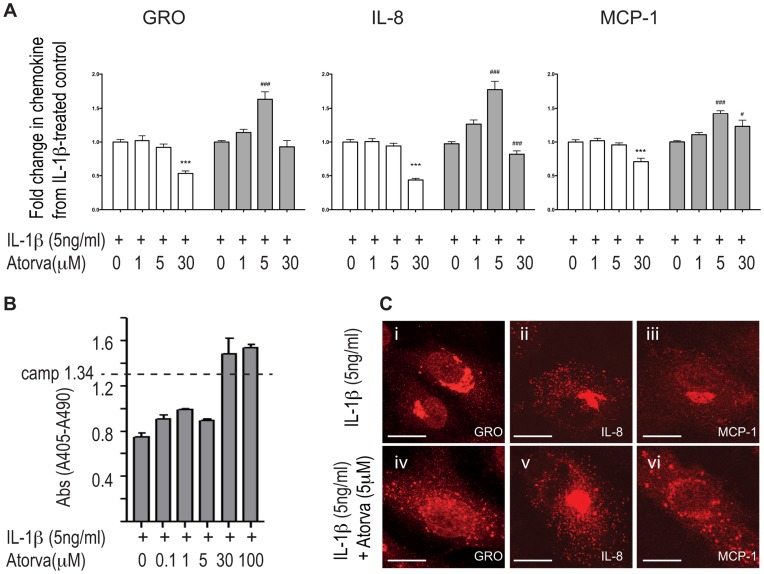
Atorvastatin induces intracellular accumulation and subcellular redistribution of IL-1β-induced chemokines in HUVECs. HUVECs were pretreated with atorvastatin before IL-1β-stimulation. Chemokine levels in supernatants (white bars) and lysates (grey bars) were analyzed by ELISA (A). Apoptosis was measured by the Cell Death Detection ELISA^PLUS^ Kit using camptothecin-treated cells (camp) as positive control (B). Immunofluorescent staining showed that GRO-α (Ci, iv), IL-8 (Cii, v) and MCP-1 (Ciii, vi) in IL-1β-stimulated HUVECs (Ci-iii) were subject to subcellular redistribution when cells were pretreated with 5 µM atorvastatin (Civ-vi). *** p<0.001 compared to supernatants from IL-1β-treated control cells. # p<0.05 and ### p<0.001 compared to lysates from IL-1β-treated control cells.

Pretreatment with moderate doses of atorvastatin (5 µM) consistently increased the intracellular levels of the IL-1β-induced chemokines GRO-α, IL-8 and MCP-1 (Figure 1A). There was no apparent apoptosis as assessed by measurement of histone-complexed DNA fragments (Figure 1B). By contrast, atorvastatin did not affect chemokine levels in the absence of IL-1β (data not shown).

### Atorvastatin Changes the Subcellular Distribution of IL-1β-induced Endothelial Chemokines

The distribution of chemokines in cells treated with IL-1β but not atorvastatin correlated well with previous reports from our and other groups [21], [22], [23]; GRO-α was predominantly present in small cytoplasmic punctae (Figure 1Ci) where it to a great extent colocalized with IL-8 and MCP-1 (data not shown). IL-8 (Figure 1Cii) was also prominently present in cigar-shaped Weibel-Palade bodies, while MCP-1 showed a similar distribution to GRO-α (Figure 1Ciii). By contrast, when exposed to atorvastatin (5 µM) chemokines were still found in these locations, but in addition, they were also observed in larger perinuclear, granules (Figure 1Civ-vi). Furthermore, atorvastatin induced similar changes in subcellular distribution of GRO-α in HAoECs treated with atorvastatin and IL-1β, and in HUVECs treated with atorvastatin and TNF-α or atorvastatin and LPS (figure S1). This indicates that our findings are transferable to aortic endothelial cells, which are the most relevant model cell type for atherosclerosis studies, and that the effect is not limited to the effect of IL-1β.

### Atorvastatin Drives Chemokine Accumulation in Endothelial Cells by Inhibiting Prenylation

**Figure 2:**
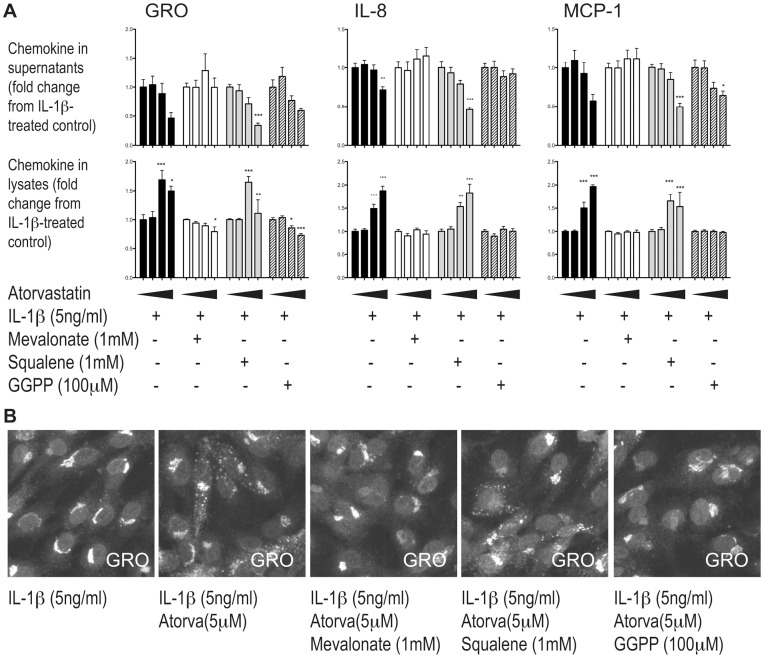
The accumulation and subcellular redistribution of IL-1β-induced chemokines in HUVECs depends on inhibition of isoprenylation. HUVECs were pretreated with atorvastatin together with medium (black bars), mevalonate (white bars), squalene (grey bars) or GGPP (hatched bars) before IL-1β-stimulation. In the histogram the four bars of the same color represent cells pretreated with increasing doses of atorvastatin (from the left: 0, 1, 5 and 30 µM). Chemokine levels were measured by ELISA for GRO-α, IL-8, MCP-1 (A) and immunostaining for GRO-α (B). * p<0.05, ** p<0.01 and *** p<0.001 from IL-1β-treated control cells without atorvastatin.

Statins inhibit mevalonate synthesis from HMG–CoA, thereby blocking the synthesis of cholesterol via squalene. To confirm the involvement of the mevalonate pathway in our experiments, we supplemented the cultures with mevalonate, observing a complete reversal of the increased lysate levels (Figure 2A) and subcellular distribution (Figure 2B and data not shown). In addition to blocking cholesterol synthesis, depletion of mevalonate simultaneously inhibits production of isoprenoids such as GGPP. Both the increase in intracellular protein and the occurrence of chemokine in endocytic compartments were prevented by supplementing cultures with GGPP, but not the cholesterol precursor squalene, (Figure 2A-B), indicating that atorvastatin exerts these effects by inhibiting isoprenylation rather than merely depleting the cells of cholesterol.

### GRO-α, IL-8 and MCP-1 are Targeted to the Same Compartment by Atorvastatin

**Figure 3:**
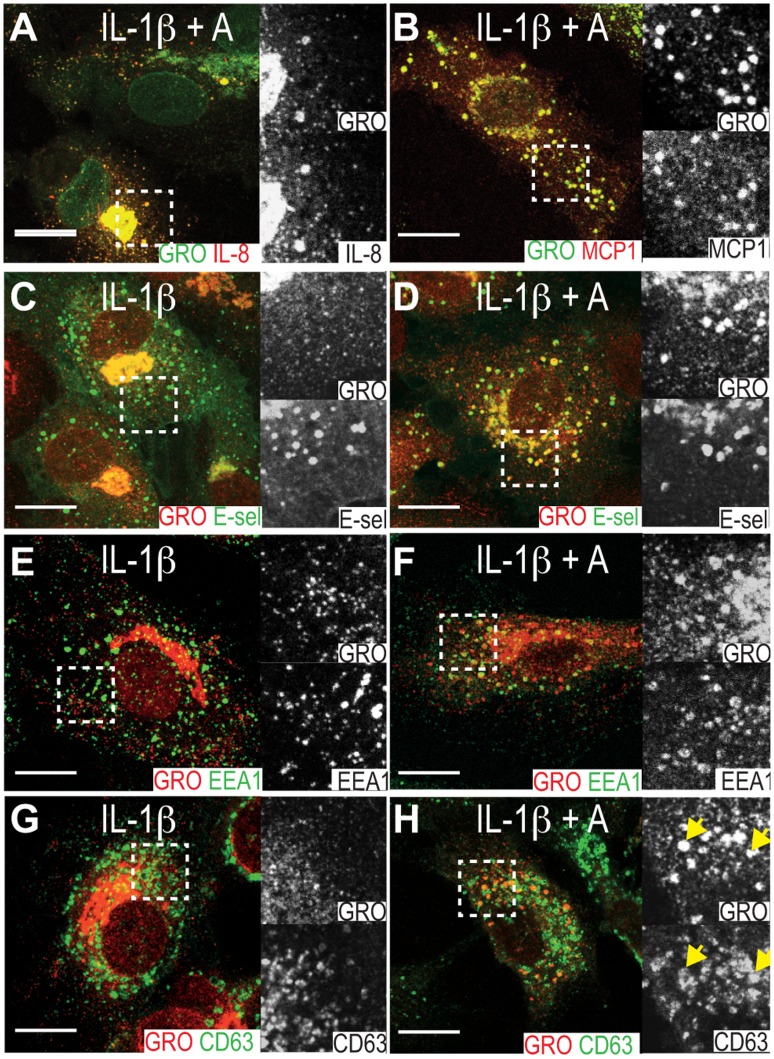
Atorvastatin induces colocalization of IL-1β-induced chemokines and E-selectin in endocytic compartments. HUVECs were pretreated with 5 µM atorvastatin (A-B, D, F, G) or medium (C, E, G) before IL-1β-stimulation, fixation and immunostaining for GRO-α (A-H), IL-8 (A), MCP-1 (B), E-selectin (C-D), EEA-1 (E-F) and CD63 (G-H). Yellow arrows in panel H outlines colocalization of GRO-α and CD63. Scale bars are 10 µm.

In HUVECs, IL-1β-induced GRO-α, IL-8 and MCP-1 colocalize in small cytoplasmic punctae probably representing endothelial type 2 granules [22], [23]. Double immunostainings for GRO-α and IL-8 or GRO-α and MCP-1 in cells pretreated with atorvastatin (5 µM) showed that the IL-1β-induced chemokines also colocalized in the atorvastatin-induced subcellular compartment (Figure 3A-B).

### GRO-α Localizes to CD63 Positive MVBs in Atorvastatin Treated Cells

**Figure 4:**
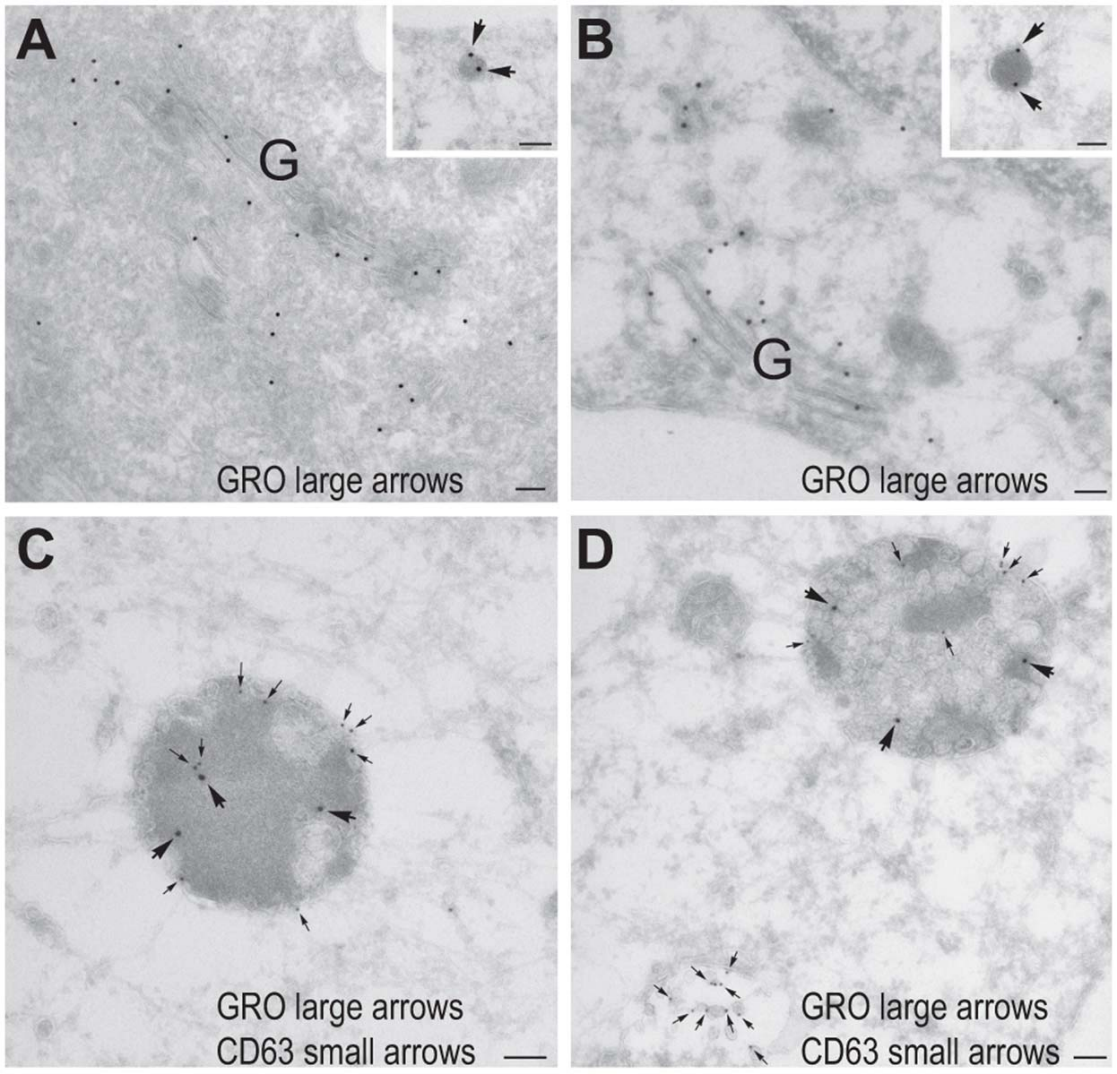
GRO-α localizes to CD63 positive multivesicular bodies in atorvastatin treated cells. Thawed cryo sections of fixed HUVECs pretreated without (A) or with 10 µM atorvastatin (B-D) were either single labeled (A-B) with anti-GRO-α antibody followed by 15 nm protein A gold (large arrows in insets), or double labeled (C-D) with anti-GRO-α antibody followed by 15 nm protein A gold (large arrows) and anti-CD63 antibody followed by 10 nm protein A gold (small arrows). Both in control cells (A) and atorvastatin-treated cells (B) labeling for GRO-α was found in the Golgi region (G) and small electron dense vesicles (insets in A and B). In atorvastatin-treated cells labeling for GRO-α was also found in large electron dense, CD63-positive multivesicular bodies (C-D). Note that the electron lucent CD63 positive multivesicular body in the lower left corner of D is GRO-α negative. Scale bars are 100 nm.

Further characterization of the atorvastin-induced compartment using GRO-α as a marker revealed that it also contained E-selectin (Figure 3C-D). This was also seen in HAoECs after stimulation with IL-1β and atorvastatin and in HUVEC after stimulation with either TNF-α or LPS together with atorvastatin (figure S1). E-selectin is an endothelial-specific adhesion molecule, whose surface expression after induction is largely regulated by the rate of endocytosis and degradation [24]. Accordingly, we costained for GRO-α and the early and late endosomal markers EEA-1 and CD63 in atorvastatin-exposed cells, observing partial colocalization with both markers (Figure 3E-H), indicating that atorvastatin targets IL-1β-induced GRO-α, IL-8 and MCP-1 to the endocytic pathway. No difference in the subcellular distribution of E-selectin was observed between control and atorvastatin-treated cells (Figure 3C-D). Immunoelectron microscopy confirmed the presence of GRO-α in the Golgi and small electron dense vesicles of approximately 100 nm, probably representing endothelial type 2 granules [22], in both control cells and atorvastatin-treated cells (Figure 4A-B). In addition, atorvastatin-treated cells showed a signal for GRO-α in large, electron dense, CD63-positive MVBs (Figure 4C-D). By contrast, another population of more electron lucent CD63-positive MVBs appeared to be negative for GRO-α (Figure 4D, lower left corner). Note that we were unable to observe GRO-α in clathrin-coated pits or bound to the plasma membrane to any great extent, perhaps indicating that GRO-α enters the endocytic pathway by lateral transport rather than endocytosis.

### Simvastatin and Fluvastatin but not the Water-soluble Pravastatin Redistribute GRO-α in the Same Manner as Atorvastatin

**Figure 5:**
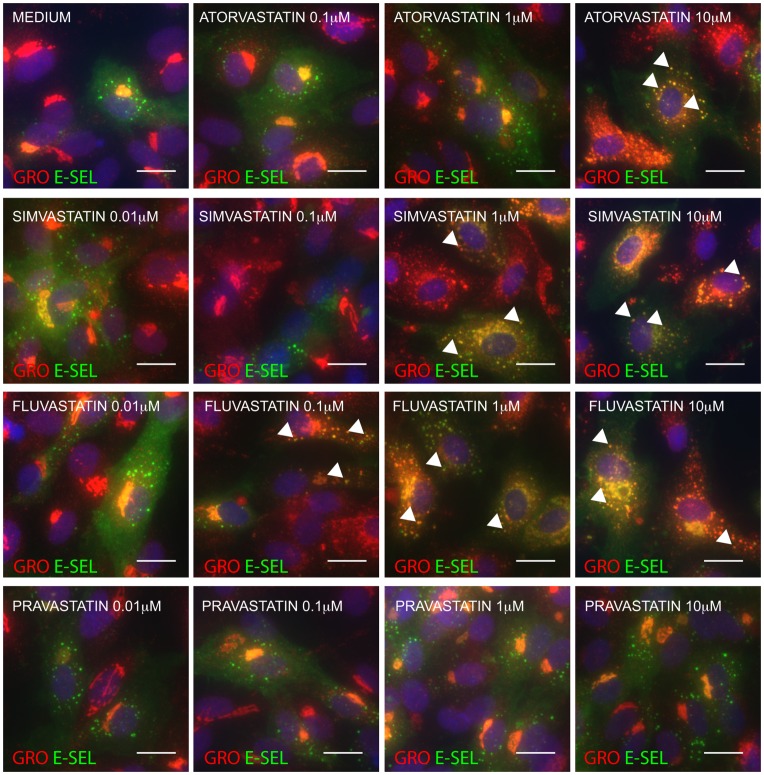
Simvastatin and fluvastatin but not pravastatin induce colocalization of GRO-α and E-selectin in the same manner as atorvastatin. HUVECs were pretreated with medium or increasing concentrations of atorvastatin, simvastatin, fluvastatin or pravastatin as indicated, before IL-1β-stimulation, fixation and immunostaining for GRO-α and E-selectin. Arrowheads show examples of colocalization between GRO-α and E-selectin. Scale bars are 10 µm.

To examine if the effect on chemokine redistribution was specific to atorvastatin, we immunostained HUVEC treated with increasing concentrations of simvastatin, fluvastatin and pravastatin before stimulation with IL-1β (Figure 5). Both simvastatin and fluvastatin caused colocalization of GRO-α and E-selectin to the same extent as atorvastatin. The water-soluble pravastatin was however unable to induce this effect at any of the concentrations tested (0.01, 0.1, 1 and 10 µM).

## Discussion

This study demonstrates for the first time that statins direct inflammatory chemokines like GRO-α, IL-8 and MCP-1 to E-selectin-containing endosomes and CD63-positive MVBs, possibly targeting the chemokines for intracellular degradation.

We observed that treatment with atorvastatin increased intraendothelial levels of GRO-α, IL-8 and MCP-1 and induced their copresence in large irregularly rounded perinuclear compartments. To describe these compartments we performed costainings for GRO-α and proteins with well-known intracellular distributions. After treatment with three different statins, IL-1β-induced GRO-α colocalized with E-selectin, a leukocyte adhesion molecule expressed by endothelial cells after inflammatory cytokine activation. E-selectin clusters in lipid rafts and clathrin-coated pits on endothelial surfaces and is internalized from the latter [25]. Our observation may therefore suggest that statin-treatment allows GRO-α, IL-8 and MCP-1 to enter the endocytic pathway (either by internalization or by lateral transport from the Golgi apparatus to endocytic compartments). Confirming such entry, the chemokine and E-selectin-containing compartments colocalized partly with the early endosomal marker EEA-1 and partly with the late endosomal marker CD63 in atorvastatin-exposed cells. Finally, immunoelectron microscopy of atorvastatin-treated HUVECs showed colocalization of GRO-α with CD63 in a population of electron dense MVBs.

Because the bulk of internalized E-selectin is trafficked to lysosomes and degraded [26], the pronounced colocalization of GRO-α with E-selectin and its presence in electron dense MVBs may suggest that the studied chemokines are also subject to lysosomal degradation in atorvastatin-treated HUVECs, however further studies would be needed to confirm if this is indeed the fate of the redistributed chemokines.

In contrast to E-selectin, GRO-α, IL-8 and MCP-1 were not present at detectable levels in endosomal compartments in the absence of statins. Endocytosis of E-selectin is known to be clathrin-dependent [25], suggesting that we may be observing a redistribution of surface-bound chemokines to clathrin-coated pits rather than a generalized effect on endocytosis. Statins have previously been shown to inhibit the inclusion of TLR4 in lipid rafts [27]. Such lipid raft modulation represents a possible mechanism by which receptor translocation into detergent-soluble membrane fractions like clathrin-coated pits may be induced. We were, however, unable to observe GRO-α in clathrin-coated pits or even bound to the plasma membrane to any great extent by immunoelectron microscopy, perhaps indicating that lateral transport from the Golgi might be the predominant route of entry for GRO-α into endosomes and MVBs rather than increased endocytosis.

While the use of HUVECs for most of our experiments may be a limitation of the present study, we partly bridge this gap by showing that atorvastatin is also able to redirect GRO-α to E-selectin-containing compartments in HAoECs, which may represent a more relevant model system in relation to statins and atherosclerosis.

Supplementation with mevalonate confirmed that the atorvastatin-induced effect was due to inhibition of HMG-CoA reductase. Mevalonate is a precursor for the synthesis of cholesterol and isoprenoids. Isoprenoids are lipophilic moieties that can be covalently linked to proteins of the small GTPase family and γ-subunits of heterotrimeric G-proteins, allowing association of the proteins with membranes. Supplementing our cultures with the isoprenoid substrate GGPP completely inhibited the accumulation of chemokines in lysates and the accumulation in the endocytic pathway, demonstrating that the effect is isoprenoid-dependent. Because of the important role of cholesterol in many aspects of cellular membrane transport, we also supplemented our cultures with the cholesterol precursor squalene. However, squalene did not prevent the atorvastatin-effect, leading us to conclude that cholesterol-depletion was not a contributing factor.

The *in vivo* relevance of our findings deserves a discussion focused on the concentrations of statins used in our study and those thought to be effective *in vivo*. Redistribution of chemokines to multivesicular bodies became prominent when atorvastatin concentrations reached 5 µM. Plasma concentrations of atorvastatin are in the range of 9 nM (5 ng/ml) after a single dose of 20 mg [28], 180 nM (100 ng/ml) after a single dose of 80 mg atorvastatin in healthy volunteers [29], and reach 450 nM (250 ng/ml) in dyslipidemic patients after longer term treatment [30]. Furthermore, a randomized, double-blind, placebo-control multicenter trial showed that the beneficial effects of statins on inflammatory parameters are observed already in patients on low-dose (20 mg daily) rosuvastatin therapy [31], where statin plasma levels are indeed likely to be much lower than 5 µM.

One should however keep in mind that plasma levels of atorvastatin are subject to great variations. Levels after a single dose of 20 mg were increased 18-fold in critically ill sepsis patients compared to healthy volunteers [28] and a range of drugs are able to interfere with hepatic metabolism of atorvastatin [30]. While most patients on atorvastatin therapy are expected to have plasma levels much lower than the concentration used in our study, it is not inconceivable that plasma levels in the range of 5 µM may be relevant in individual patients on high-dose atorvastatin therapy. Furthermore, simvastatin and fluvastatin were able to relocate GRO-α to E-selectin-containing compartments at 1 and 0.1 µM concentrations, respectively, which are in the upper region of concentrations measured in plasma from patients on a treatment regime of 40 mg once daily [30].

Further studies would be needed to address if this phenomenon occurs in patients treated with statins, and whether the accumulation of chemokines in the endocytic pathway has an anti-inflammatory effect due to chemokine sequestering, thus representing an extension of the presumably beneficial effect of statin-treatment seen at lower plasma levels [31], or opposed to this, if such an accumulation could be detrimental to vascular function.

In conclusion, our findings show that atorvastatin is able to retain inflammatory chemokines in endothelial E-selectin-containing endosomes and CD63-positive MVBs, potentially targeting them for intracellular degradation. How such retainment and presumable breakdown of chemokines affects endothelial cell behavior under proinflammatory activation and in the context of atherosclerosis development remains to be seen.

## Supporting Information

Figure S1 **Atorvastatin induces colocalization of GRO-α and E-selectin in aortic endothelial cells and in HUVEC treated with TNF-α and LPS.** HAoEC (C-12271, Promocell) (A) and HUVEC (B-C) were pretreated with atorvastatin before stimulation with IL-1β (A), TNF-α (B) or LPS (C), fixation and immunostaining for GRO-α and E-selectin. Scale bars are 10μm.
(PDF)
