# Supplementary material for: Statins Affect the Presentation of Endothelial Chemokines by Targeting to Multivesicular Bodies
Source: PLoS One. 2012 Jul 16;7(7):e40673. doi: 10.1371/journal.pone.0040673 (PMC3398041; doi:10.1371/journal.pone.0040673)
Supplement: Figure S1 — Atorvastatin induces colocalization of GRO-α and E-selectin in aortic endothelial cells and in HUVEC treated with TNF-α and LPS. HAoEC (C-12271, Promocell) (A) and HUVEC (B-C) were pretreated with atorvastatin before stimulation with IL-1β (A), TNF-α (B) or LPS (C), fixation and immunostaining for GRO-α and E-selectin. Scale bars are 10μm. (PDF) [file pone.0040673.s001.pdf]

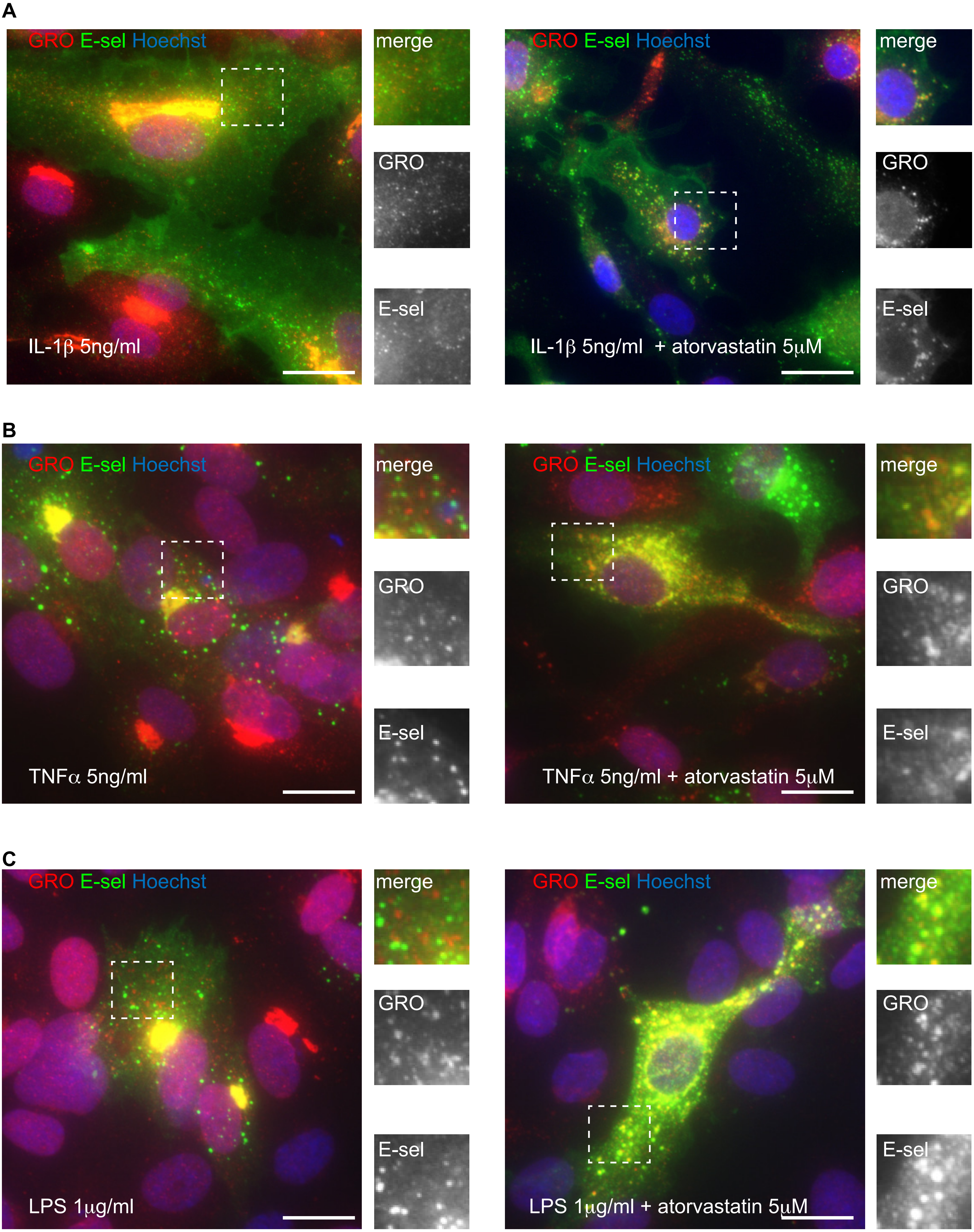

Supplementary figure 1: HAoEC (C-12271, Promocell) (A) and HUVEC (B-C) were pretreated with atorvastatin before stimulation with IL-1β (A), TNFα (B) or LPS (C), fixation and immunostaining for GRO-α and E-selectin. Scale bars are 10μm.
